# Organic semiconductor/graphene oxide composites as a photo-anode for photo-electrochemical applications†

**DOI:** 10.1039/c8ra06546b

**Published:** 2018-10-22

**Authors:** Farheen Khurshid, M. Jeyavelan, M. Sterlin Leo Hudson, Samuthira Nagarajan

**Affiliations:** Department of Chemistry, Central University of Tamil Nadu Thiruvarur India snagarajan@cutn.ac.in +91-9443046272; Department of Physics, Central University of Tamil Nadu Thiruvarur India msterlinleo@cutn.ac.in +91-9486860214

## Abstract

An intimate physical mixture of graphene oxide (GO) and semiconducting organic molecules like bromophenathrene (BrPh) and bromopyrene (BrPy) was prepared by using a ball milling technique. The structural, microstructural, physical and chemical properties of the mixtures (20 wt% of GO) were analyzed by X-ray diffraction, SEM, FT-IR, TGA and TCSPC studies. Furthermore, the electrochemical properties like AC electrical conductivity, transient photocurrent response (PCTR) and open circuit voltage (OCVD) of the samples were analyzed. It has been observed from TCSPC and OCVD measurements that 20 wt% of GO in the semiconductor composite leads to an enhanced life-time of photo-generated charge carriers. The physical mixture composites exhibit a higher photocurrent than pure BrPh and BrPy.

## Abstract 2

New organic materials with longer life-times of the charge carrier and enhancement of photocurrent generation were developed.

## Graphical abstract

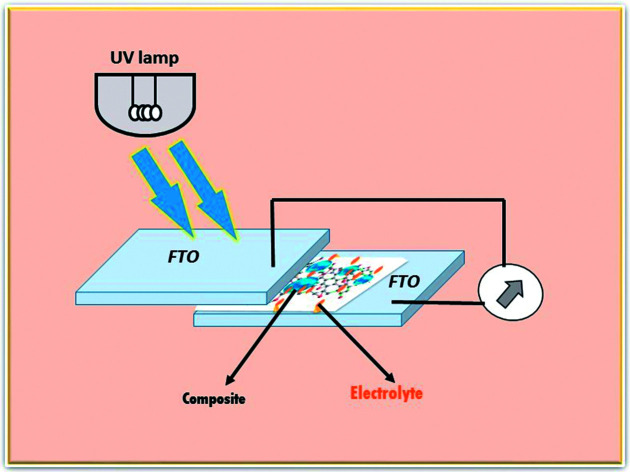

## 1. Introduction

Recent advancement in renewable energy technology for harnessing solar energy through a clean and environmental friendly approach has gained significant research interest in exploring efficient photovoltaic materials. The wide bandgap (>3 eV) inorganic semiconductors such as TiO_2_, ZnO and SnO_2_ have been used widely in solar cells,^1–3^ photocatalytic cells^4–6^ and other optoelectronic devices.^7–9^ In particular in dye sensitized solar cells, the TiO_2_/ZnO metal oxide semiconductor composite is generally used as a photo anode^1^ for collecting the photo generated charge carriers from the sensitizer. It also serves as a sensitizer in photocatalytic cells.^4^ However, the issues associated with metal oxide semiconductors are the higher charge carrier recombination rate,^10^ and poor electrical conductivity.^11,12^ In order to decrease the charge carrier recombination rate in the metal oxide semiconductor researchers have used GO and reduced GO (rGO) along with oxide semiconductors in mechanical mixing.^13,14^ Graphene owing to its high electrical conductivity,^15^ high carrier mobility,^16^ high power density,^17^ large surface areas (∼2600 m^2^ g^−1^),^18^ sustainability^19,20^ and roll-to-roll compatibility,^21^ offers an ideal choice for enhancing the electronic properties of optoelectronic materials when forming composites.^22,23^ Azarang *et al.*^24^ have observed that ZnO/rGO composite (1.7 wt% rGO) shows enhanced photocurrent generation of 1.22 mA cm^−2^, which is three times higher than that observed for pristine ZnO. Sookhakian *et al.*^25^ have found ZnS/rGO composite shows higher photocurrent generation of 1.9 μA cm^−2^. Recent reports suggest that rGO or GO can act as a medium for fast carrier transportation between the semiconductor (sensitizer) and FTO substrate. Since the observed photo generated charge carrier's life time in the rGO or GO based semiconductor composite is much longer than that of pristine semiconductor, which increases the photocurrent generation and thereby suppressing the recombination rate of the sensitizer.^13,14^ Recently, organic semiconductors such as polymers and small organic molecules find tremendous applications towards optoelectronic devices.^26,27^ In organic semiconductor devices, the metal oxide semiconductors such as TiO_2_, ZnO and SnO_2_ were replaced by flexible organic semiconducting materials having high electrical conductivity (higher than that of metal oxide semiconductors),^28–30^ Furthermore, the optoelectronic properties of the organic semiconductors were improved by functionalization or by making composite with GO/rGO. Zheng *et al.*^14^ reported that the photo generated charge carrier transport kinetics in organic semiconductor, poly(3-hexylthiophene) has been significantly improved when GO and rGO (acceptor) was added.

In this work, we have chosen small organic semiconductor donor molecules such as 1-bromopyrene (BrPy) and 9-bromophenanthrene (BrPh) mixed with GO to understand the photo exited charge carrier transport kinetics between the donor and acceptor species. The organic semiconductor/GO composite was prepared under solvent free condition by using ball milling. The donor (organic semiconductor) and acceptor species (GO) were mixed in the ratio of 4 : 1 and the photo-excited carrier transport kinetics of as prepared composites (BrPh/GO (20 wt%) and BrPy/GO (20 wt%)) were investigated.

## 2. Experimental section

### 2.1 Materials

Graphite powder (200 mesh) was purchased from Alfa Aesar and 1-bromoPyrene (BrPy), 9-bromophenanthrene (BrPh) were procured from Sigma Aldrich.

### 2.2 Sample preparation of organic semiconductor/GO

**Fig. 1:**
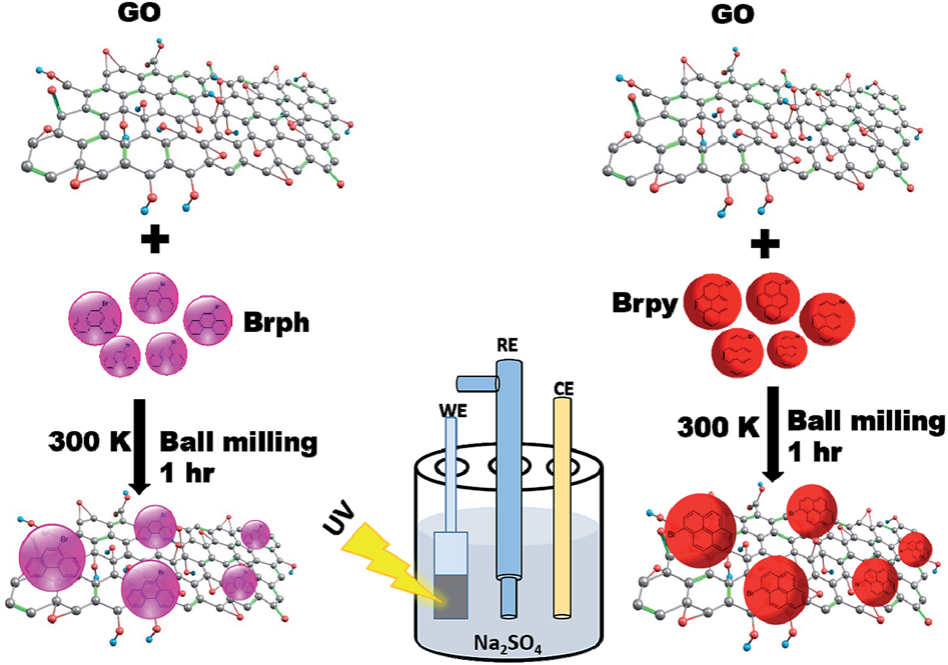
Schematic diagram represents the synthesis of physical mixture composites for photo electrochemical analysis.

The Fig. 1 shows the schematic representation of organic semiconductor/GO composites preparation and its photocurrent analysis. The photoactive composites were prepared through ball milling technique using FRITSCH planetary ball miller. For milling, 4 g of organic semiconductor (BrPh or BrPy) and 1 g of as prepared graphene oxide (given in ESI†) were placed in 80 ml stainless steel mixing jar and stainless steel milling balls having 5 mm diameter were used for grinding. The jar was agitated by using the planetary ball mill at 400 rpm for 60 minutes at room temperature. To prevent powder sticking to the balls and the jar walls, methanol was added as a process control agent. The obtained composites were used for electrochemical analysis.

### 2.3 Structural, microstructural, optical, thermal and electrochemical analysis

The structural analysis of the composites was carried out by using powder X-ray diffractometer (PANalytical X'Pert PRO) equipped with X'Celerator position sensitive detector using Cu Kα radiation of wavelength, *λ* = 1.5401 Å. Microstructural analysis of the samples was characterized by using JEOL scanning electron microscope (SEM). Fourier transform infrared (FT-IR) transmittance spectra was carried out using Agilent-Cary630 FT-IR spectrophotometer. The differential scanning calorimetry (DSC) and thermogravimetric analysis (TGA) of the samples were analyzed between 40 °C and 400 °C at a constant heating rate of 10 °C min^−1^ under nitrogen atmosphere (flow rate of 20 ml min^−1^) by using PerkinElmer STA 8000 TGA analyzer. The cyclic voltammetry (CV) studies were carried out by using commercial three electrodes method (WE, CE and RE) kept in 1 M of phosphate buffer solution (PBS) as supporting electrolyte. The sample was pasted on a glassy carbon electrode surface (1 × 1 mm) and dried at 40 °C and then subjected for CV measurements in the potential range of −2 to 1 V at a constant scan rate of 20 mV s^−1^.

The electrochemical impedance spectra (EIS), photocurrent transient response (PCTR) and open circuit voltage decay (OCVD) measurements were done by using BioLogic VSP-300 electrochemical workstation with the three-electrode configuration (working, reference and counter electrode). The working electrode was prepared by coating the composite film on FTO substrate (having sheet resistance of 15 Ω sq^−1^) by drop casting method (by mixing 50 mg of photo active composite with 1 ml of ethanol) and then the films were subsequently heated at 50 °C for 30 minutes using a hot plate. The active area of the composite films was found to be 3.5 cm^2^. The standard calomel electrode and platinum wire was used as a reference and counter electrode, respectively. A 0.1 M of Na_2_SO_4_ solution was used as an electrolytic medium.

## 3. Results and discussion

### 3.1 Structural, microstructural and thermal analysis

**Fig. 2:**
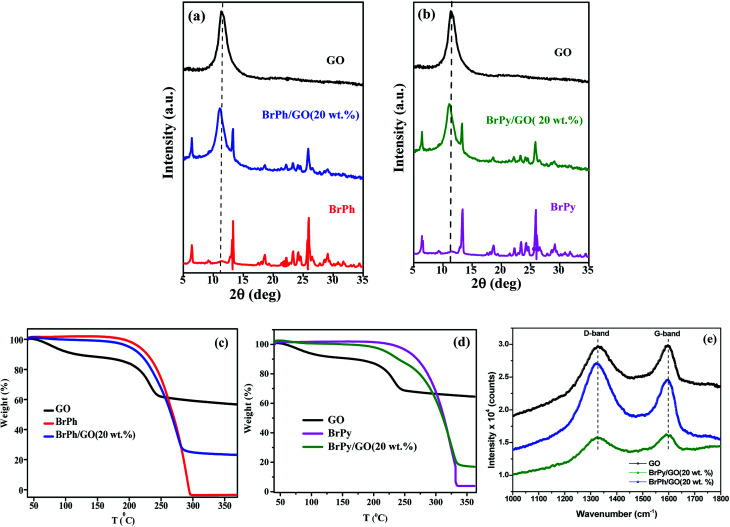
XRD diffractograms of (a) GO, BrPh and BrPh/GO composites and (b) GO, BrPy and BrPy/GO composites, (c) & (d) TGA curves of GO, BrPh/GO and BrPy/GO, (e) Raman spectra of GO, BrPh/GO and BrPy/GO.

The structural properties of the samples were characterized by using X-ray diffraction and FTIR (functional groups analysis was discussed in the ESI†) studies. Fig. 2(a) and (b) represents, the X-ray patterns of GO, BrPh/GO and BrPy/GO. The amount of GO in both the composite mixture is 20 wt%. After oxidation of graphite, the characteristic diffraction peak observed at the diffraction angle 10.8° corresponds to (002) plane for GO with the *d*-spacing of 0.77 nm. This *d*-spacing is larger than that of graphite (0.33 nm)^31^ due to the intercalation of oxygen containing functional groups between the carbon planes. Furthermore, we have observed the GO peak along with other diffraction peaks corresponding to BrPh and BrPy in the composites confirms the formation of physical mixture. Compared to pristine GO, we have observed a slight shift (Δ*d* = 0.02 nm) in the position of the GO peak towards the lower angle for both BrPh/GO and BrPy/GO, which might be due to the mixing of organic semiconductor molecules (BrPh and BrPy) with the GO or due to the ball milling.

**Fig. 3:**
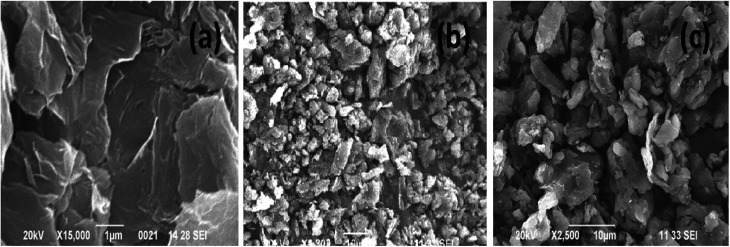
SEM images of (a) GO, (b) BrPh/GO and (c) BrPy/GO.

Fig. 2(c) and (d) displays the TGA curves of the samples. The TGA curve of GO shows that, it was thermally less stable and it started to loss its weight even at below 100 °C, due to the removal of surface adsorbed impurities (water). On further heating from 100 to 250 °C, we have observed a major weight loss of 24%, due to the pyrolysis of labile oxygen-containing functional groups.^32^ It has been observed from Fig. 2(c), a complete decomposition of BrPh occurred at around 300 °C. Whereas, BrPh/GO composite shows nearly 80% weight loss at 300 °C, suggests the decomposition of BrPh molecules in the composite. Fig. 2(d) shows that BrPy decompose completely at 335 °C. Whereas, the BrPy/GO composite shows around 80% weight loss at 335 °C due to the presence of BrPy molecules. When comparing both BrPy/GO and BrPh/GO composites with the pristine organic semiconductors (BrPy and BrPh.), the decomposition temperature of the composite is slightly lower than pristine BrPy and BrPh. This indicates that the thermal stability of the composites is altered by the addition of GO, probably due to the mixing.^33^ A minor weight loss was observed below for both BrPy/GO and BrPh/GO composite below 200 °C, suggesting the removal of surface adsorbed impurities and oxygen functionalities from GO. To understand the interaction between the GO and organic semiconductor molecules, Raman spectral analysis were carried out for BrPh/GO, BrPy/GO and pristine GO, the corresponding spectra are shown in Fig. 2(e). The representative Raman spectra of GO, BrPh/GO, BrPy/GO shows two prominent bands which are found at 1325 and 1597 cm^−1^ known as D-band and G-band respectively. Generally, the G-band arises due to the doubly degenerate zone-center E_2_g mode vibration of carbon atoms and D-band originated due to the formation sp^3^ hybridized carbon structure.^39^ Both G-band and D-band are also observed in the Raman spectra of BrPh/GO and BrPy/GO confirming the existence of GO in the composite. Scanning electron microscopic analysis reveals the surface morphologies of GO, BrPh/GO and BrPy/GO shown in Fig. 3. It has been observed from the Fig. 3(b) and (c), after addition of the organic semiconductor with GO leads to the morphological defects.

### 3.2 Photo-electrochemical analysis (EIS, PCTR and OCVD)

**Fig. 4:**
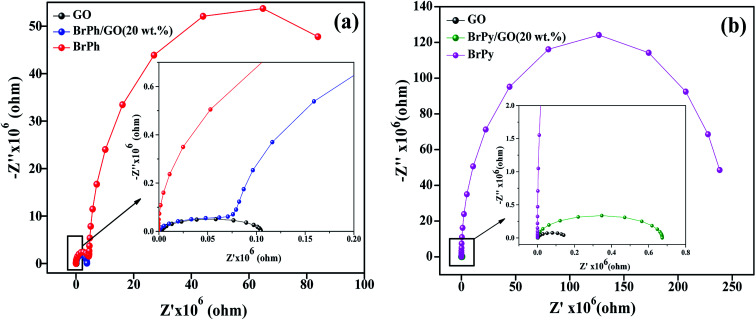
Impedance curves of (a) BrPh/GO composite and (b) BrPy/GO composite (inset figure represents the magnified impedance curve of at lower frequency region).

The electrochemical impedance curves of the composite films were recorded between 500 mHZ to 1 MHZ at ambient temperature. The impedance properties of the composites are shown in the Fig. 4(a) and (b). The Nyquist curves were plotted against real parts (*Z*′) *vs.* imaginary parts (−*Z*′′) of the impedances. It has been observed from Fig. 4(a), the BrPh and BrPh/GO displays the impedance curve which consists two semicircles, the smaller one (shown as an inset figure in Fig. 4(a)) in the high frequency region corresponding to the charge transfer resistance (*R*_ct_) at the interface between the working electrode and electrolyte and larger one in the low frequency region corresponding to the ohmic resistance of the film.^34^ As from Fig. 4(b), we have observed the impedance curve with a single semicircle (shown as an inset figure in Fig. 4(b)), corresponding to the charge transfer resistance (*R*_ct_) at the interface between the working electrode and electrolyte. As observed from the impedance curve of BrPh/GO and BrPy/GO, the radius of the semicircles is reduced dramatically after the addition of GO. It might be due to the presence of various functional groups, which adsorbs large number of ions from the electrolyte, as a result a better charge transportation between the working electrode and electrolyte. Hence, GO enhancing both ionic adsorption and charge carrier transportation in the composites film. Therefore, the smaller charge transfers resistance, leads to the better transportation of photo generated charge carriers (in semiconductor), which will enhance the photo conversion efficiency of the electrochemical solar cell (dye sensitized solar cell) and capacitance of the electrochemical capacitors (super capacitors). The AC electrical conductivity of the composite film can be calculated from the following equation;1
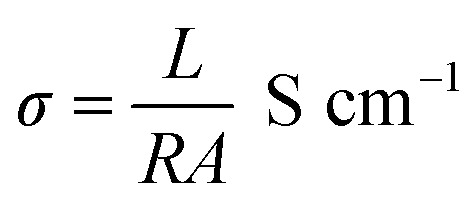
where, *σ* is the AC conductivity of the composites, *R* is resistance which is calculated from the *Z*-fit, *L* is the length of the film and *A* is the cross-sectional area of the film. The BrPh/GO and BrPy/GO exhibits approximately 1000 times higher AC conductivity than BrPh and BrPy, respectively. The calculated impedance curve values are given in the Table 1.

**Table 1:** AC electrical conductivity of BrPh, BrPy, BrPh/GO and BrPy/GO composite

Samples	BrPh	BrPy	BrPh/GO	BrPy/GO
AC conductivity (S cm^−1^)	1.37 × 10^−9^	3.5 × 10^−10^	1.62 × 10^−6^	1.05 × 10^−7^

**Fig. 5:**
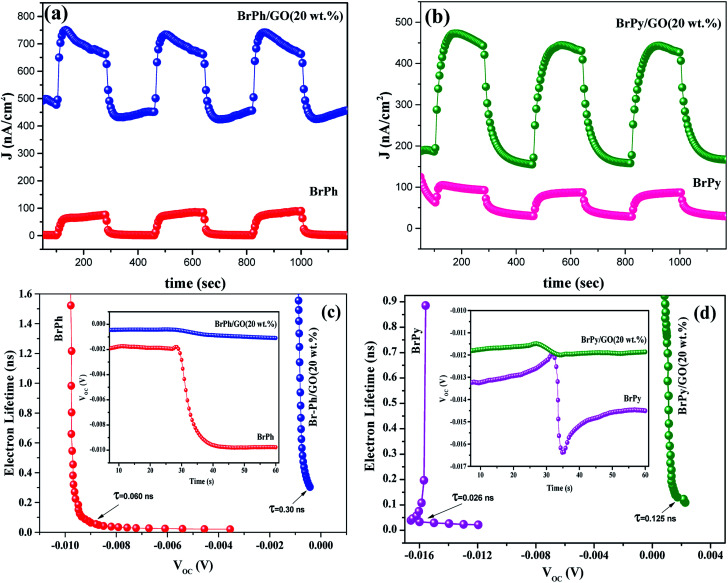
(a) PCTR of BrPh/GO composite, (b) PCTR of BrPy/GO composite, (c) carrier lifetime of BrPh/GO composite and (d) carrier lifetime of BrPy/GO composite (inset of graph represents the respective OCVD measurement).

**Fig. 6:**
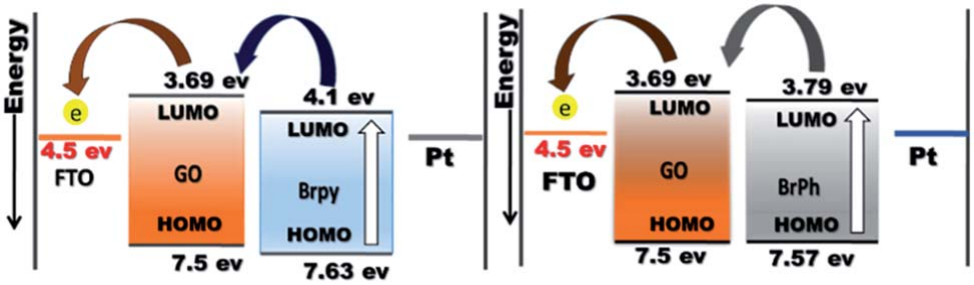
Schematic diagram for charge transport mechanism.

The optoelectronic properties such as photocurrent generation, open circuit voltage and carrier recombination can be investigated from the photocurrent transient response (PCTR) and open circuit voltage decay measurements (OCVD). In order to explore the photocurrent generation and open circuit voltage properties of the composites, the photo-anode (working electrode) was exposed under UV irradiation (300 W) with ON/OFF cycles at an interval of 5 minutes. The PCTR measurements were recorded with 10 mV input supply between working electrode and reference electrode and the photocurrent between working and counter electrode was monitored as a function of time. The measured photocurrent curves for BrPh/GO and BrPy/GO composites are shown in Fig. 5(a) and (b), respectively and the calculated photocurrent, dark current values are given in the Table 2. As observed from Fig. 5(a) and (b), once UV light illuminated on the photo-anode, the photocurrent was increased rapidly and it achieved the saturation. It has been observed from Fig. 5(a), the composites show enhanced photocurrent properties than that of pristine BrPh and BrPy. The photocurrent of BrPh/GO is found to increases from 75 to 273 nA cm^−2^ after addition of GO. The enhanced photocurrent generation of BrPh/GO composite is approximately 4 times higher than pristine BrPh. On the other hand, the BrPy/GO composite film coated photo-anode exhibits a photocurrent generation of 287 nA cm^−2^ (Fig. 5(b)), which is approximately 8 times higher than that of pristine BrPy. The reason behind the improved photocurrent generation is that, beside the light absorption, the transportation and separation of photo exited charge carrier are also considered to be the key factors which can improve the photocurrent generation. Graphene is an excellent choice carrier for transportation due to its 2D π-conjugation structure. Therefore, a fast transportation of photo generated charge carrier occurs from semiconductor into graphene *via* percolation mechanism.^35^ Many researchers have reported that, the photocurrent generation of the semiconductor can be improved by the addition of rGO or GO.^36,37^ Zheng *et al.*^14^ have observed that, a better charge transportation occurs between P3HT and GO which leads to the enhanced photocurrent performance of P3HT/GO composite compared to that of P3HT/rGO. Therefore, in the present study, the photo exited electrons from the organic semiconductor was effectively trapped by the GO and thereby reducing the recombination probability. Furthermore, as discussed in the EIS analysis, the low charge transfer resistance between composite film (working electrode) and electrolyte also place a major role in improving the photocurrent properties of the composites. The calculation of HOMO and LUMO was discussed in the ESI (Section 4).† As observed from the Fig. 6, the LUMO of the GO (3.69 eV) is found to be near to the LUMO of BrPh (3.79) and BrPy (4.1 eV). This suggests that, the photo generated charge carriers in the semiconductors can immediately transfered from its LUMO to the LUMO of GO. So that, the photo generated charge carriers can stay longer time before recombination. The calculated lifetime of photo-induced charge carrier by the time correlated single photo counting measurements (Section-3 of ESI†), which reveals the carrier lifetime in BrPh/GO (0.1538 ns) and BrPy/GO (0.14082 ns) was slightly improved than that of pristine semiconductors (0.066 and 0.0670 ns).

**Table 2:** PCTR measurement data of BrPh, BrPh/GO, BrPy and BrPy/GO

Sample	On/off min	Photocurrent density (nA cm^−2^)	Dark current density (nA cm^−2^)
BrPh	5	75	2
BrPh/GO	5	273	477
BrPy	5	34	60
BrPy/GO	5	287	189

Therefore, the probability of carrier recombination has been reduced in the semiconductor by the addition of 20 wt% GO. Additionally, the BrPh/GO composite shows high dark current density (477 nA cm^−2^) than the pristine BrPh (2 nA cm^−2^). Similarly, the BrPy/GO composite exhibits high dark current density (189 nA cm^−2^) than pristine BrPy (60 nA cm^−2^). The likely reason for high conduction of dark current both BrPh/GO and BrPy/GO composites has already discussed in AC impedance analysis.

The open circuit voltage decay of the photo anode was analyzed by monitoring the potential growth by switching on the UV for 30 seconds and potential decay by switch off UV for 30 seconds. The open circuit voltage decay (OCVD) measurements are also confirmed the enhanced charge carrier lifetime of the composites. Basically, the OCVD is used for the measurement of photo-induced electron recombination property.^38^ The electron recombination kinetics were investigated by monitoring the *V*_OC_ as a function of time after illuminating the light for 30 second. The OCVD and carrier lifetime curves of BrPh, BrPh/GO, pristine BrPy and BrPy/GO composites were shown in Fig. 5(c) and (d). It has been observed from the inset figures of Fig. 5(c), a sharp decay in the *V*_OC_ occurred from −0.002 to −0.010 V and the corresponding life-time of photo-generated charge carriers (*τ*_n_ = 0.060 ns) was calculated from the OCVD curve for BrPh. Similarly, from inset of Fig. 5(d), the carrier lifetime (*τ*_n_ = 0.026 ns) of BrPy was measured from a sharp decay in *V*_OC_ from −0.013 to −0.015 V. Whereas, the BrPh/GO exhibits a small gradual decrease from −0.0003 to −0.0009 V and the BrPy/GO displays the similar gradual decrease from −0.0114 to −0.0119 V, the corresponding carrier life times (*τ*_n_ = 0.30 and 0.125 ns) for both composites were calculated from the OCVD curves shown in Fig. 5(c) and (d). The carrier lifetime of the sample was calculated by using the following equation:^39^2
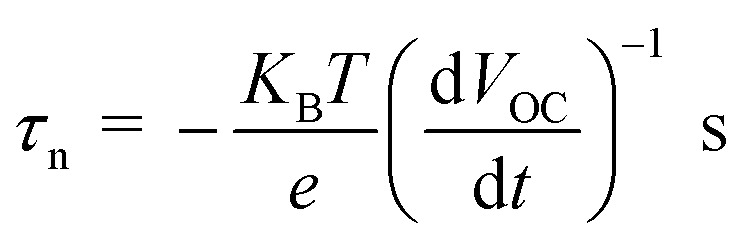
where, *K*_B_ – Boltzmann constant, *T* – temperature in Kelvin, *e* – charge of electron, d*V*_OC_/d*t* – slope, which is calculated from the OCVD curve.

The above results reveal that the enhanced lifetime of photo-generated charge carriers of composites (BrPh/GO and BrPy/GO) leads to the high photocurrent generation than that of pristine BrPh and BrPy. Furthermore, the high carrier life time of the composites is attributed to the slower recombination rate of photo-generated charge carriers and hence more electron might be surviving for the back reaction. This superior phenomenon is useful to enhance the photocurrent generation in DSSC. Moreover, photo-current from the photo active semiconductor film (pristine organic semiconductor) is determined by the speed of exited electrons withdrawn from the semiconductor to conducting electrode (FTO) and the recombination at electrolyte/film interface.

## 4. Conclusions

Organic semiconductor/GO composite was prepared by solvent free mechanical milling. When compared with the pristine BrPh and BrPy, the physical mixture composites BrPh/GO and BrPy/GO exhibits nearly 1000 times higher AC conductance. The increased AC conductance of composite signifies that, the presence of 20 wt% GO has increased the charge separation efficiency. The transient photocurrent response measurements reveal that, the composites show higher photocurrent generation than pristine semiconductors, which a result, the 20 wt% of GO in the composite has increased the lifetime of the photo generated charge carriers. Hence, this increase in life time of photogenerated charge carriers in composites reduces the recombination rate, which was confirmed by the TCPSC and OCVD measurements, making it an ideal choice for photo-electrochemical applications.

## Conflicts of interest

There are no conflicts to declare.

## Supplementary Material

RA-008-C8RA06546B-s001
