# Supplementary material for: Organic semiconductor/graphene oxide composites as a photo-anode for photo-electrochemical applications
Source: RSC Adv. 2018 Oct 22;8(63):35959–65. doi: 10.1039/c8ra06546b (PMC9088551; doi:10.1039/c8ra06546b)
Supplement: RA-008-C8RA06546B-s001 [file RA-008-C8RA06546B-s001.pdf]

Electronic Supplementary Material (ESI) for RSC Advances.
This journal is © The Royal Society of Chemistry 2018

Supporting information

Organic Semiconductor/Graphene Oxide Composites as a Photo-Anode for Photo-Electrochemical Applications

Farheen Khurshid,[a] M. Jayavelan,[b] M. Sterlin Leo Hudson[b*] and Samuthira Nagarajan[a*]

Department of Chemistry, Central University of Tamil Nadu. Thiruvarur – 610 005, India.

Department of Physics, Central University of Tamil Nadu. Thiruvarur – 610 005, India.

**Corresponding author Email: snagarajan@cutn.ac.in (SN), msterlinleo@cutn.ac.in (MSLH)*

1. Synthesis of Graphene Oxide (GO)

Graphene oxide was prepared by modified Hummer's method. [1] About 5g of graphite powder and 2.5 g of $NaNO_3$ in 108 ml of H_2SO_4 and 12 ml of H_3PO_4 was stirred in an ice-cooled bath for 10 min. Then, 15 g of $KMnO_4$ was gradually added into the above mixture at 5 ˚C under constant stirring. The suspension was then first allowed to react for two hours in ice bath and then one hour at 40 ˚C. Afterwards, the temperature of the reaction was raised and maintained at 98 ˚C for 1 hour. To control the net reaction, 400 ml of deionized (DI) water was added to the suspension. After 5 min, 15 ml of H_2O_2 was added. Finally, the reaction product was filtered with G4 sintered crucible and washed repeatedly with deionized water and 5% HCl solution, until the suspension becomes neutral (pH = 7). The final product was collected and dried at 60 ˚C in a hot air oven.

2. FT-IR Analysis

Fig.S1 (a) & (b) shows, the FTIR spectra of GO, BrPy, BrPy/GO, BrPh and BrPh/GO. The spectral peak observed at 3145 cm^{-1} which corresponds to the O-H stretching vibrations, the peak at 1717 cm^{-1}, which is corresponds to C=O stretching vibration groups, 1366 cm^{-1} corresponding to the C-OH stretching vibration, the peak at 1216 cm^{-1} corresponds to C-O stretching vibrations and then the peak at 1612 cm^{-1} associated with skeletal vibration of unreacted graphitic domain in GO.[2] Thus, all vibrational peaks confirms, the successful synthesize of GO from Graphite. Whereas, the vibrational peaks of pristine organic semiconductors were observed in FTIR spectra, the sharp absorption peaks at 708 cm^{-1}, 1183 cm^{-1} and 747 cm^{-1} are corresponding to the out of plan-vibration of the three adjacent C-H bonds of the BrPy & BrPh. [3] In addition to that the strong peak found at 838 and weak peaks at 1430 and 1592 are associated with C=C bonds of BrPy & BrPh. Spectra of composite consist both vibrational peaks of parent compounds. The FTIR spectra of the ball milled composite consist both vibrational peaks of parent compounds and there was no new peak has been observed for the composite, which indicating that, there was no chemical bonding between the organic semiconductor molecule (BrPy & BrPh) and GO.

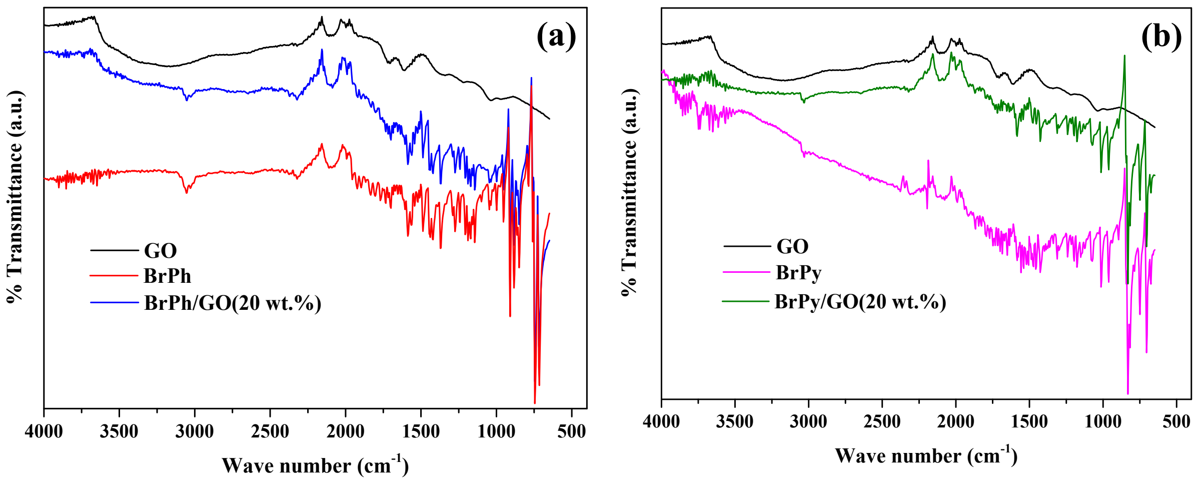

Fig. S1 (a)&(b). FTIR spectra of BrPh/GO and BrPy/GO composite, respectively.

3. Time Correlated Single Photon Counting (TCSPC)

To study the relaxation kinetics of photo induced charge carrier's in BrPh, BrPh/GO, BrPy and BrPy/GO, the time correlated single photon counting technique was employed. The samples BrPh/GO and BrPy/GO composites were excited by using a laser sources of wavelength 300 and 340 nm, respectively and the corresponding lifetime of exited state was monitored. The obtained decay kinetics are shown in Fig.S2 (a) & (b). The carrier lifetime was calculated from the bi-exponential fit on the experimental decay spectra of the samples and fitted parameters are given in the (Table-1). On comparing with pristine semiconductors (BrPh and BrPy), the GO composite semiconductors, BrPh/GO and BrPy/GO exhibits higher carrier life-time. This significant improvement in the life-time of photo generated charge carriers are due to the presence of 20 wt.% GO in the composites. The photo generated charge carriers in the semiconductor were relaxed in the intermediate state of GO (LUMO) due to its carrier acceptor property, which leads to the fast photo induced carrier transport. Life time analysis of the samples were given in Table-1.

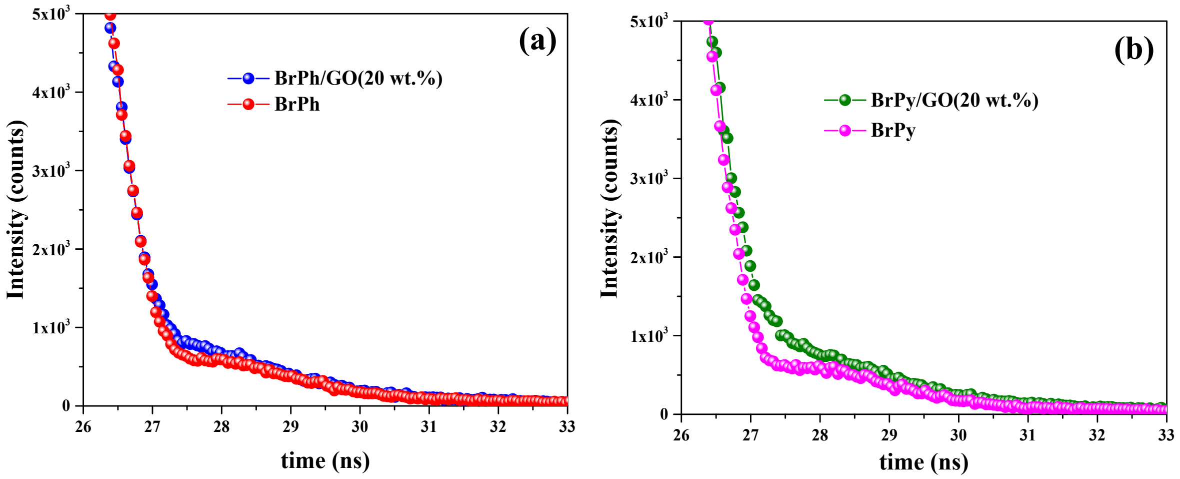

Fig. S2 Fluorescence decay spectra of (a) BrPh/GO composite and (b) BrPy/GO composite.

Table-1 Lifetime analysis of the samples.

Sample	A_1	τ_1 (ns)	A_2	τ_2 (ns)	$<\tau>$ (ns)
BrPh/GO	-58.45	0.1532	58.37	0.1543	0.1538
BrPh	-151.9	0.0062	0.204	0.3047	0.066
BrPy/GO	-41.54	0.1400	41.41	0.1416	0.14082
BrPy	645.2	0.0066	0.1214	0.4204	0.0670

4. HOMO/LUMO Calculation

The optical band gap (E_{opt}) of the GO, BrPh and BrPy were calculated from the UV absorbance spectra by using $(\alpha h\gamma)^2$~ ($h\gamma$-E_{opt}) where, α is the absorption coefficient. The respective UV absorbance spectra is shown in Fig.S4 (a) & (b). The calculated E_{opt} values of GO, BrPh and BrPy samples are 3.81, 3.78 and 3.47 eV, respectively. The band broadening of the samples resulting from the localized states were calculated from the Urbach tail (E_u) which obeys $\alpha=\alpha_0\exp(h\nu/E_u)$. Therefore, the reciprocal of the slope obtained from the linear fitting of ln α vs hγ plot is referred as E_u (shown in Fig.S4(c)) and the obtained E_u values of GO, BrPh, BrPy are 1.20, 0.227, 0.098, respectively. From the above results, the energy gap ($E_g = E_{opt} - E_u$) between the HOMO/LUMO determined for GO, BrPh and BrPy are 3.81, 3.78 and 3.47 eV, respectively. In order to estimate the reduction potential, the samples were analyzed by the cyclic voltammetry. Fig.S4(d) displays the cyclic voltammetry curves of BrPh, BrPy, GO and Phosphate buffer solution (PBS). It has been observed that, the reduction peaks of BrPh, BrPy and GO were founded at -1.31, -0.33 and -0.81 V, respectively. Reports suggest that the reduction peak corresponding to the electrochemical reduction of oxygen groups in GO can be observed between 0.7 to 1.2 V. [4]

Furthermore, the HOMO and LUMO of the samples were calculated from the following equations; [4]

$$E_{LUMO} = E^{onset}_{reduction} - E^{onset}_{reduction\left(Fe(CN)_6^{3-/4-}\right)} + 4.8 \quad eV \qquad (2)$$

$$E_{HOMO} = E_{LUMO} + E_g \quad eV \qquad (3)$$

The onset reduction potentials were calculated from the CV curves of the samples, whereas, the well-known onset reduction potential of $K_3[Fe(CN)_6]$ is 0.3 V. The estimated HOMO/LUMO energies are shown schematically in Fig.3.

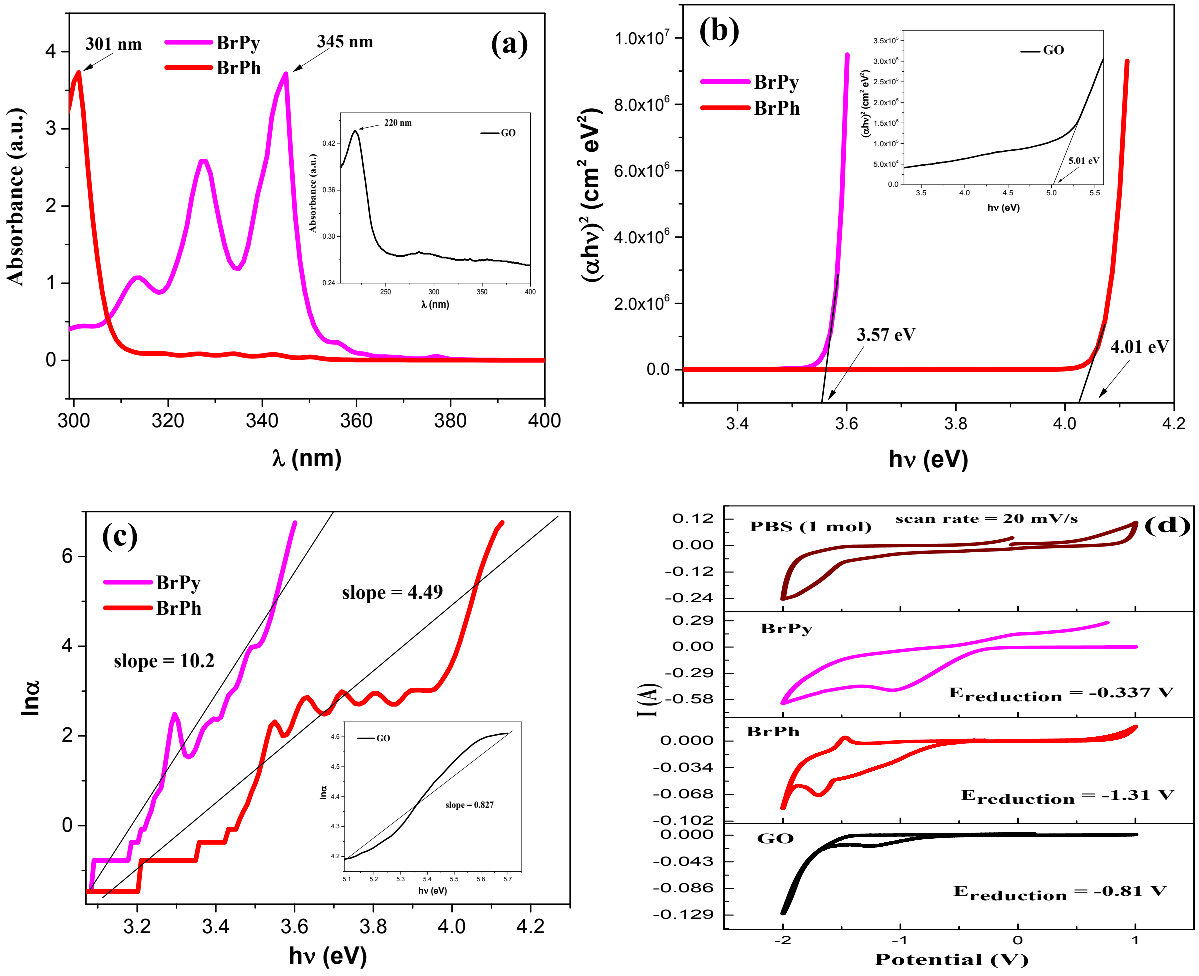

Fig.3(a) UV absorbance spectra, (b) Tauc plot, (c) Urbach tail measurement plots of BrPy, BrPh and GO (shown in the insert figure of respective figures) and (d) cyclic voltammetry curves of PBS, GO, BrPy and BrPh.

References

1. F. Khurshid, M. Jeyavelan, K. Takahashi, M. S. L. Hudson and S. Nagarajan, *RSC Advances*, 2018, **8**, 20440-20449.
2. N. Kumar, S. Das, C. Bernhard and G. Varma, *Superconductor Science and Technology*, 2013, **26**, 095008.
3. L. Zhang, C. Li, A. Liu and G. Shi, *Journal of Materials Chemistry*, 2012, **22**, 8438-8443.
4. F. Zheng, W.-L. Xu, H.-D. Jin, X.-T. Hao and K. P. Ghiggino, *RSC Advances*, 2015, **5**, 89515-89520.
